# Intraluminal valves: development, function and disease

**DOI:** 10.1242/dmm.030825

**Published:** 2017-11-01

**Authors:** Xin Geng, Boksik Cha, Md. Riaj Mahamud, R. Sathish Srinivasan

**Affiliations:** 1 Cardiovascular Biology Research Program, Oklahoma Medical Research Foundation, Oklahoma City, OK 73104, USA; 2 Department of Cell Biology, University of Oklahoma Health Sciences Center, Oklahoma City, OK 73104, USA

**Keywords:** Wnt/β-catenin signaling, Calcific aortic valve disease, Lymphatic vasculature, Mechanobiology, Valves

## Abstract

The circulatory system consists of the heart, blood vessels and lymphatic vessels, which function in parallel to provide nutrients and remove waste from the body. Vascular function depends on valves, which regulate unidirectional fluid flow against gravitational and pressure gradients. Severe valve disorders can cause mortality and some are associated with severe morbidity. Although cardiac valve defects can be treated by valve replacement surgery, no treatment is currently available for valve disorders of the veins and lymphatics. Thus, a better understanding of valves, their development and the progression of valve disease is warranted. In the past decade, molecules that are important for vascular function in humans have been identified, with mouse studies also providing new insights into valve formation and function. Intriguing similarities have recently emerged between the different types of valves concerning their molecular identity, architecture and development. Shear stress generated by fluid flow has also been shown to regulate endothelial cell identity in valves. Here, we review our current understanding of valve development with an emphasis on its mechanobiology and significance to human health, and highlight unanswered questions and translational opportunities.

## Abstract (precis)

**Summary:** This Review summarizes the similarities between cardiac and vascular valves, and highlights unanswered questions in diagnosis and treatment of valve disorders.

## Introduction

The survival of multicellular organisms depends on the ability of their cells to receive nutrients and to dispose of waste. In vertebrates, two interconnected vascular networks (blood and lymphatic) meet this basic requirement. Arteries (excluding the pulmonary artery) in the systemic circulation carry oxygenated blood from the heart to tissues and organs, and additionally provide them with glucose and other nutrients derived from the small intestine. These arteries undergo a series of branching events to generate small-diameter capillaries called arterioles. Owing to high hydrostatic pressure and low oncotic pressure (see Box 1 for a glossary of terms), arterioles release water and other small molecules, such as glucose and albumin, into the interstitial space, which are then taken up by cells. Cells release waste materials, such as lactic acid and carbonic acid, into the interstitial space, which are mostly taken up by venules, very small veins that progressively fuse to form larger veins that return deoxygenated blood to the heart (Breslin, 2014; Levick and Michel, 2010; Wiig and Swartz, 2012). Low hydrostatic pressure and high oncotic pressure inside the venules is important for interstitial fluid reabsorption. Approximately 10% of the interstitial fluid is left behind by the venules, which is absorbed by lymphatic vessels and returned to the venous blood circulation. Defects in this fluid transportation system can lead to stagnation of blood or interstitial fluid, resulting in vascular thrombosis or edema, respectively (Box 1).

**Box 1. Glossary**

**Bicuspid aortic valve disease:** condition in which people are born with only two flaps in the aortic valve rather than the usual three; occurs in ∼1% of humans.

**Calcification:** a pathological condition of the cardiac valves in which the valvular interstitial cells acquire bone-cell-like characteristics. The valves become rigid, resulting in their inability to open and close properly.

**Cardiac cushion:** specialized cells within the endocardial tube in the developing heart that generate the valves and septum (the wall dividing the left and right sides of the heart).

**Chylothorax:** accumulation of lymph in the thoracic cavity (which encapsulates the heart and lungs) due to defective or damaged lymphatic vessels.

**Chylous ascites:** accumulation of lymph in the abdominal cavity due to defective or damaged lymphatic vessels.

**Diastole:** the phase of the heartbeat during which the heart muscles relax.

**Edema:** accumulation of water and molecules of small molecular mass, resulting in the swelling of the interstitial tissue.

**Emberger syndrome:** a disease caused by mutations in the zinc-finger transcription factor GATA2. Symptoms include lymphedema and leukemia.

**Embolism:** blockage of blood vessels by an object. When vessels are blocked by a blood clot, the condition is called thromboembolism.

**Hydrops** **fetalis:** a condition caused by the accumulation of fluid in developing embryos.

**Hydrostatic pressure:** pressure exerted on the walls of blood or lymphatic vessels by the weight of the fluid.

**Intraluminal pressure:** pressure exerted on the walls of blood or lymphatic vessels by the weight of the fluid and the pressure generated by fluid flow.

**Laminar shear stress:** frictional force experienced by the endothelial cells due to the orderly flow of blood or lymph. Straight portions of blood or lymphatic vessels experience such a flow pattern.

**Lymphedema:** edema caused by defective lymphatic vascular functioning.

**Lymphedema-distichiasis syndrome:** a genetic disorder caused by mutations in the forkhead-domain transcription factor FOXC2. Symptoms include lymphedema, two rows of eyelashes and, rarely, heart defects.

**Oncotic pressure:** pressure caused by a higher protein concentration within blood capillaries than in the extracellular milieu. Higher oncotic pressure pulls water into the capillaries.

**Oscillatory shear stress:** frictional force experienced by the endothelial cells due to the disorderly flow of blood or lymph. Branch points of blood or lymphatic vessels experience such a flow pattern.

**Stenosis:** an abnormal narrowing of valves due to their inability to open completely.

**Systole:** the phase of the heartbeat during which the heart muscles contract.

**Valve vegetations:** abnormal growth of clots in the cardiac valves. Such vegetations could be caused by bacterial infections, inflammation or metastatic tumors.

**Venous insufficiency:** stagnation of blood within the veins in the lower extremities due to the inefficient functioning of venous valves.

**Venous ulcers:** wounds caused by stagnation of blood within the veins. Such wounds normally occur on the lower legs.

In mammals, four main types of valve form to regulate the unidirectional flow of fluid in different organs – cardiac valves (in the heart), venous valves (VVs; in veins), lymphatic valves (LVs; in the lymphatic vessels) and lymphovenous valves (LVVs; at the sites where lymph is returned to blood circulation) (see below). When the development or functioning of these valves fail or deteriorate, it can result in morbidity and death. Thus, new therapeutic approaches to prevent valve deterioration are urgently needed.

Here, we provide an overview of how valves contribute to health and disease, and review recent findings that highlight the similarities that exist between aortic valves (a type of cardiac valve) and vascular valves (LVs, VVs and LVVs). We discuss the mechanisms by which shear stress regulates these commonalities. We propose that, by exploring these similarities, we may be able to uncover the mechanisms that govern the central nature of valves. Finally, we speculate on how these findings could provide opportunities to diagnose and treat valve disorders.

## Mammalian valves: an overview

In this section we provide an overview of the main types of mammalian valves and the diseases that are caused by defects in these structures.

### Cardiac valves

The mammalian heart has four valves: the aortic and pulmonic valves (known as the semilunar valves), and the mitral and tricuspid valves (known as the atrioventricular valves). Defects in any one of these valves can have serious consequences (Kim and Ruckdeschel, 2016; Nkomo et al., 2006; Shah and Raney, 2008). Calcification is a pathological condition that causes stiffening (stenosis; Box 1) of the cardiac valves due to changes in the composition of the extracellular matrix (ECM). The aortic valve is the most prone to this disorder. Calcific aortic valve disease (CAVD) is the most common valve disease in the developed world, where an estimated 2- to 4-million people suffer with this disease (Yutzey et al., 2014). Approximately 15,000 deaths per year are attributed to CAVD in North America, and this number is expected to increase rapidly owing to the aging population and the lack of prevention strategies (Lindman et al., 2016). Calcified aortic valves cannot open fully during systole or close fully during diastole (Box 1) (Demer and Tintut, 2008; Yutzey et al., 2014). Aortic valve stenosis causes the left ventricle to work harder to meet the metabolic demands of the body, resulting in left-ventricular hypertrophy, which increases the risk of heart attack and stroke (Lindman et al., 2016).

Prosthetic valves have significantly reduced the mortality associated with cardiac valve disorders. However, patients need long-term treatment with blood thinners to prevent clot formation on the valves, which are associated with uncontrolled bleeding, which itself could cause severe morbidity (Dangas et al., 2016; Nishimura et al., 2017). Additionally, prosthetic valves have a limited lifespan and might need replacement or readjustment with time (Nishimura et al., 2017). Hence, research is ongoing to better understand aortic valve disease and to develop approaches that will stop/slow its progression.

### Venous valves

Owing to low intraluminal pressure (Box 1), blood transport within the veins (frequently against gravitational pressure) depends on VVs, the degeneration of which wreaks havoc on normal vascular physiology. Defective VVs cause primary chronic venous insufficiency (Box 1), which leads to elevated venous pressure, edema and pooling of the blood. According to a Scottish study, around 6-10% of the general population, and >20% of those over 50 years old, are estimated to have some form of chronic venous insufficiency (Ruckley et al., 2002; Weber et al., 2016). In the US, chronic venous insufficiency is the seventh leading cause of chronic debilitating disease (Meissner et al., 2007b; Weber et al., 2016). The severity of this disease could be mild (spider veins), moderate (varicose veins) or severe (edema, venous eczema and venous ulcers) (Box 1) (Meissner et al., 2007b). The most serious forms of chronic venous insufficiency can cause necrotic ulcers that may require limb amputations (Tsai et al., 2005).

Owing to their architecture, the downstream side (behind the valves with respect to the direction of blood flow) of VVs are prone to blood stasis (Brooks et al., 2009). Primary chronic venous insufficiency causes increased blood pooling within the veins, which results in hypoxia, clotting and endothelial cell inflammation, otherwise known as deep vein thrombosis (DVT). Complications of DVT include pulmonary embolism (Box 1), due to clots that dislodge from VVs and migrate to the lungs, and secondary chronic venous insufficiency, due to inflammation permanently damaging VVs (Meissner et al., 2007a). Every year, an estimated 2-3 individuals per 10,000 will develop DVT in the developed world (Fowkes et al., 2003). In those older than 60, this number increases to nearly 10 in 10,000. Venous malformations, long-distance flights, sedentary lifestyle and hip or pelvic surgery increase the chances of developing DVT (Kyrle and Eichinger, 2005).

Blood thinners, which can cause bleeding, are commonly used to prevent and treat DVT (Hirsh and Hoak, 1996). Venous insufficiency in peripheral veins [such as saphenous veins (the two main veins in the leg)] is treated by laser ablation of the vein. In contrast, there is no treatment for insufficiency within central veins [such as the iliac vein (in the pelvis) or inferior vena cava (running behind the abdominal cavity to the heart)]. Valve replacement therapy is currently being explored for central veins and might become available in the future (Weber et al., 2016). VV allografts (the transplantation of valves from living donors or cadavers) are showing promising results in clinical and pre-clinical trials (Burkhart et al., 1997; Dalsing et al., 1999).

### Lymphatic and lymphovenous valves

Lymph is collected by lymphatic capillaries and transported via collecting lymphatic vessels (Davis et al., 2012). LVs within the collecting lymphatic vessels regulate unidirectional lymph flow. Ultimately, lymph collected from the body returns to the blood circulation via four LVVs, which are bilaterally located at the junction of the jugular and subclavian veins, in the neck (Geng et al., 2016; Srinivasan and Oliver, 2011). Unlike cardiac valves and VVs, the functioning of which can be imaged by color Doppler imaging, it is not currently possible to non-invasively image and quantify LV functioning in humans (Mellor et al., 2011). As a result, the significance of LV defects to human lymphedema (Box 1) is not fully understood. However, a recently developed, non-invasive ultrasound approach to imaging LVVs might yield quantitative evidence regarding the importance of LVVs to lymphatic vascular physiology (Seeger et al., 2009).

Several mouse models that have defective LVs or LVVs exhibit lymph reflux, lymphedema or chylothorax (Box 1) (Bazigou et al., 2009; Geng et al., 2016; Kanady et al., 2011, 2015; Kazenwadel et al., 2015; Kriederman et al., 2003; Martin-Almedina et al., 2016; Munger et al., 2017; Petrova et al., 2004). In one study, the severity of LV defects in the thoracic duct of a mouse model [*connexin-37* (*Cx*)*37^−/−^;Cx43^+/−^*; see further details in Vascular valve disorders, below] was shown to correlate with the onset of chylothorax and postnatal death (Kanady et al., 2011). Additionally, overexpression of vascular endothelial growth factor C (VEGFC) in adipocytes resulted in the incompetence of LVs (likely due to the dilation of lymphatic vessels) and in chylothorax (Nitschke et al., 2017). These reports validate the importance of LVs and LVVs in lymphatic vascular physiology. According to current models, defective valves cause lymph to stagnate in the collecting vessels; the increased pressure is transmitted upstream to the capillaries, inhibiting lymph uptake and exacerbating lymphatic vascular defects, such as lymphedema and chylothorax (Davis et al., 2012).

In summary, defects in the cardiac valve, VVs, LVs or LVVs could cause severe morbidity or mortality. However, the etiology of valve disorders is not fully understood. In the following section, we discuss the current understanding that underscores the involvement of genetic factors in the initiation and progression of these diseases.

## Genetic contributions to valve disorders

### Calcific aortic valve disease

Age, hypertension, kidney disease, high dietary fat, smoking and a sedentary lifestyle are implicated in CAVD (Yutzey et al., 2014). In addition, polymorphisms in genes that regulate lipid metabolism, inflammation and bone formation have been reported in patients with CAVD (Guauque-Olarte et al., 2015; Thanassoulis et al., 2013; Yutzey et al., 2014). For example, heterozygous mutations in *NOTCH1* cause bicuspid aortic valve disease (Box 1) and individuals with these mutations are prone to develop CAVD (Garg, 2006; Garg et al., 2005; LaHaye et al., 2014) (Table 1). Notch signaling is thought to antagonize the differentiation of aortic valve cells into bone-like cells (Acharya et al., 2011).

**Table 1.**
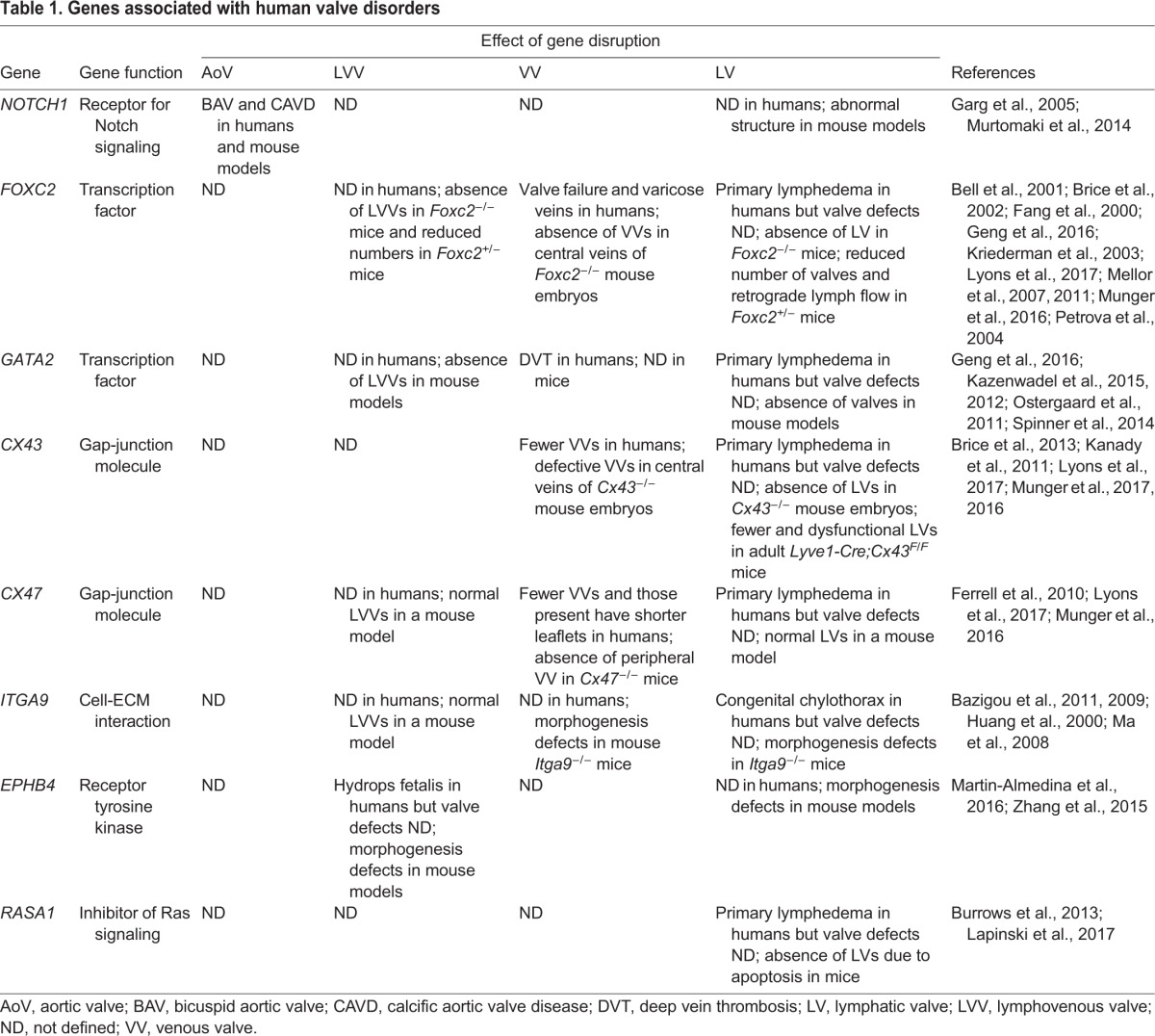
**Genes associated with human valve disorders**

### Vascular valve disorders

Varicose veins and chronic venous insufficiency have a strong familial association (Krysa et al., 2012), but only a few genes have been linked to these diseases. In contrast, numerous gene mutations are associated with lymphedema or chylothorax in human patients (Brouillard et al., 2014). We discuss here only those genes that are known to be critical for vascular valve development in mouse models (Table 1).

#### FOXC2

Dominant heterozygous mutations in the forkhead family transcription factor FOXC2 are associated with human lymphedema, venous insufficiency and varicose veins (Bell et al., 2001; Brice et al., 2002; Fang et al., 2000; Lyons et al., 2017; Mellor et al., 2007, 2011). In patients with venous insufficiency and varicose veins, VVs function abnormally and result in a retrograde blood flow pattern, as revealed by Doppler imaging (Brice et al., 2002; Mellor et al., 2007). Recently, lymphedema patients with *FOXC2* mutations were also found to possess fewer VVs, and those present had shorter leaflets (Lyons et al., 2017).

*Foxc2^+/−^* mice, which are models for lymphedema-distichiasis syndrome (Box 1), display retrograde lymph flow (Kriederman et al., 2003), possibly because they have incompetent LVs. *Foxc2^+/−^* embryos have 50% fewer LVs in their dorsal skin (Kanady et al., 2015); whether the remaining LVs are functional remains unknown. Interestingly, LV numbers are not reduced in the mesentery of *Foxc2^+/−^* embryos, implying a tissue-specific requirement for *Foxc2* dosage during LV development (Kanady et al., 2015). Loss of one *Foxc2* allele causes a variable LVV phenotype in mice; whereas some *Foxc2^+/−^* embryos have no LVVs and develop severe edema, others have one LVV at the junction of the jugular and subclavian veins (instead of the normal two LVVs) (Geng et al., 2016).

#### GATA2

Dominant heterozygous mutations in the zinc-finger transcription factor GATA2 are found in individuals with Emberger syndrome (Box 1) (Ostergaard et al., 2011). Approximately 30% of patients carrying mutations in *GATA2* develop lymphedema (*n*=14 from 8 families). Another study reported lymphedema with incomplete penetrance in 3 out of 10 patients with *GATA2* mutations (Kazenwadel et al., 2012). In another cohort of 57 patients with *GATA2* mutations, 11% developed lymphedema (Spinner et al., 2014).

A total of 25% of patients with *GATA2* mutations in the above cohort developed thrombotic events, such as DVT, pulmonary embolism, portal vein thrombosis and catheter-related thrombosis (Spinner et al., 2014), possibly because *GATA2* deficiency in the endothelium causes coagulopathy. The VVs of these patients were not analyzed.

Mice lacking *Gata2* in vascular endothelial cells lack LVVs and LVs (Geng et al., 2016; Kazenwadel et al., 2015). Whether GATA2 is necessary for VV development is currently unknown.

#### *CX43*

Mutations in the gap-junction molecule CX43 are observed in human lymphedema patients (Brice et al., 2013). *Cx43^−/−^* mouse embryos have no mesenteric LVs (Kanady et al., 2011). The conditional deletion of *Cx43* in the lymphatic vasculature using *Lyve1-Cre* results in the formation of fewer and dysfunctional LVs in postnatal and adult mice (Munger et al., 2017).

CX43 is also important for VV development. The central VVs are either absent or defective in *Cx43^−/−^* embryos (Munger et al., 2016). Whether peripheral VVs require CX43 for their formation is currently unknown because *Cx43^−/−^* mice die soon after birth and peripheral VVs develop postnatally (Bazigou et al., 2011; Munger et al., 2016; Reaume et al., 1995). Mice with a conditional deletion of *Cx43* in endothelial cells, using *Tie2-Cre*, are viable (Liao et al., 2001; Theis et al., 2001). Whether these mice possess peripheral VVs remains to be investigated.

#### CX47

Mutations in *CX47* are observed in human lymphedema patients (Ferrell et al., 2010; Lyons et al., 2017), who have fewer and shorter VVs (Lyons et al., 2017). *Cx47^−/−^* mice lack VVs in most peripheral veins, but possess LVVs, LVs and VVs in central veins (such as those located in the jugular vein) (Munger et al., 2016). When compared with *Cx43^−/−^* littermates, double-null mutants for *Cx47* and *Cx43* have more severe VV defects in the central veins, indicating that these two connexins may have overlapping roles in VV development. Interestingly, *Cx47* expression is downregulated in the LVs of *Lyve1-Cre;Cx43^f/f^* embryos, indicating that *Cx47* expression might also depend on CX43 (Munger et al., 2016).

#### ITGA9

Mutations in integrin-α9 (*ITGA9*) were identified in human fetuses with congenital chylothorax (Ma et al., 2008). *Itga9^−/−^* mice recapitulated this phenotype and died soon after birth with chylothorax (Huang et al., 2000). These embryos lack LVs and VVs (Bazigou et al., 2011, 2009), but LVVs appear to develop normally in these mutants (Hess et al., 2014).

#### EPHB4

Autosomal dominant mutations in the receptor tyrosine kinase ephrin type-B receptor 4 (EPHB4) were identified in families with a history of lymphatic-related hydrops fetalis (Box 1) (Martin-Almedina et al., 2016). Morphogenesis of LVVs is defective in mice lacking *EphB4* (Martin-Almedina et al., 2016). Using function-blocking antibodies, it was reported that EPHB4 is necessary for the development of LVs (Zhang et al., 2015).

#### RASA1

Loss-of-function mutations in the Ras GTPase RASA1 belong to a large group of diseases known as the rasopathies, in which the Ras signaling pathway is hyperactivated (Brouillard et al., 2014). A subset of patients with rasopathies display lymphedema, chylothorax or chylous ascites with variable penetrance (Box 1) (Brouillard et al., 2014). *RASA1* mutations are associated with lymphedema in a subset of carriers (Burrows et al., 2013). Deletion of *Rasa1* from adult mice results in the progressive deterioration of LV function due to apoptotic cell death of valvular endothelial cells (Lapinski et al., 2017).

In summary, mutations in certain genes appear to predispose individuals to cardiac and vascular valve disorders (Table 1). A better understanding of their action might provide us with new opportunities for clinical intervention. Therefore, we next discuss the mechanisms of valve development and subsequently the roles of the above-mentioned genes in this developmental process.

## The stepwise development of valves

### Cardiac valves

The heart undergoes complex morphogenesis during development and these changes are intimately connected to the formation of cardiac valves. In this Review, we provide a simplistic model of heart valve development in the mouse and we refer readers to more in-depth reviews for additional details (Lin et al., 2012; Person et al., 2005).

**Fig. 1.**
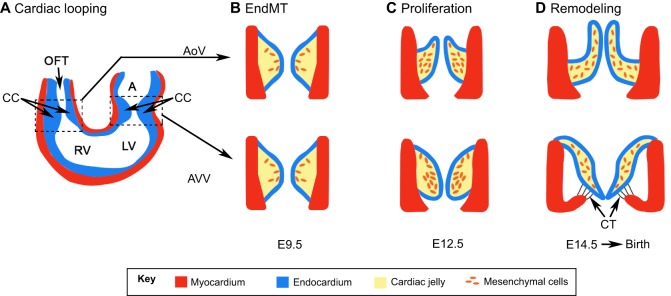
**Stepwise development of cardiac valves in mice.** (A) The heart initially forms as a simple tube with an inner endocardial (blue) and outer myocardial (red) layer. The atrium (‘A’) is yet to separate into the left and right chambers by septation, and the ventricle is a single chamber [the future left (LV) and right (RV) ventricles are depicted]. The outflow tract (OFT) has yet to separate into the pulmonary artery and dorsal aorta. The cardiac cushions (CCs) form at the atrioventricular (right box) and RV-OFT (left box) junctions. Mitral and tricuspid valves develop from the atrioventricular cushion and are collectively known as the atrioventricular valves (AVVs). The aortic (AoV) and pulmonic valves develop from the RV-OFT cushion and are collectively known as semilunar valves. For simplicity, the development of an AoV and an AVV is shown in B-D. (B) During endothelial-to-mesenchymal transition (EndMT), the endocardial cells gain a mesenchymal-cell-like characteristic and migrate into the CC. These cells are known as valvular interstitial cells (orange cells). (C) Valvular interstitial cells proliferate and secrete ECM proteins, which results in the bending of primitive valves along the direction of blood flow (arrows). (D) Valves undergo further remodeling as they mature. Fibrous chordae tendineae (CT), which distinguish AVVs from semilunar valves, attach the AVVs to the ventricular wall. Panel A was adapted with permission from Person et al. (2005); panels B-D were adapted with permission from Lin et al. (2012).

**Fig. 2.**
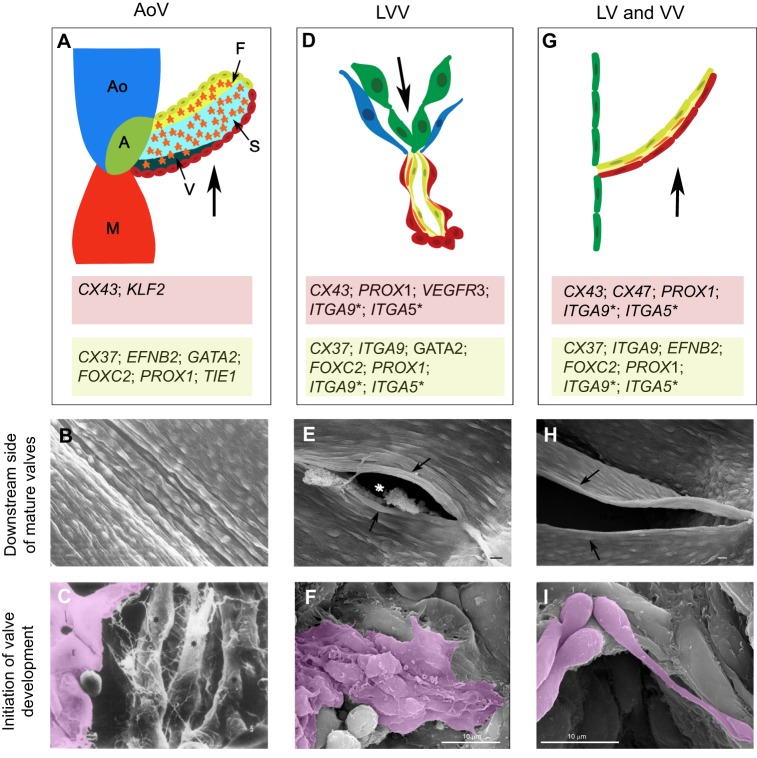
**Structural and molecular features of intraluminal valves.** (A) Schematic of a single leaflet of the aortic valve (AoV). Ao represents the aorta, M represents the myocardial layer of the left ventricle and A represents the annulus, which is a fibrous ring that supports the AoV. The small arrows point to the three inner layers of the valve: ventricularis (V), spongiosa (S) and fibrosa (F). The orange stars represent the ECM-producing activated valvular interstitial cells. The nucleated cells are the endothelial cells of the AoV. The thick arrow indicates the direction of blood flow from the left ventricle to the aorta. The endothelial cells directly facing the blood flow (upstream side) are in red and their expression profile is presented in the red box. The endothelial cells on the downstream side of the AoV are in yellow; their expression profile is presented in the light green box. (B) Scanning electron micrograph (SEM) of the downstream side of the AoV of a dog, showing the elongated morphology of the endothelial cells. Image reproduced with permission from Deck (1986). The sample is approximately 270 µm in length (left to right). (C) SEM of endothelial-to-mesenchymal transition (EndMT) occurring within a developing aortic valve of rats. Endocardial cells (E, magenta) give rise to ECM-producing valvular interstitial cells (*). Gaps are observed between the endocardial cells (arrow). The picture is a 2600× magnification of the sample. Image reproduced with permission from Markwald et al. (1975). (D) Schematic of a lymphovenous valve (LVV). The arrow indicates the direction of lymph flow. Green cells represent the lymphatic endothelial cells (LECs) of the lymph sac, blue cells represent the venous endothelial cells, red cells the LVV-forming endothelial cells (LVV-ECs), yellow cells the specialized LECs on the upstream side of LVVs, and orange cells, mural cells that lie in between the upstream and downstream sides of LVVs. The expression profiles of LVV-ECs on the downstream side of LVVs and the LECs on the upstream side of LVVs are presented in the red and light green boxes, respectively. (E) SEM of the downstream side of a mature LVV from a newborn mouse pup, showing the elongated architecture of LVV-ECs (arrows). The asterisk shows the opening through which lymph is drained from the lymph sac (located behind the plane of this image) into the veins. Image reproduced with permission from Geng et al. (2016). (F) SEM of LVV-ECs (magenta) delaminating into the lumen of the embryonic veins in an E12.0 mouse embryo. The cells pile on top of each other and form filopodia-like projections. Image reproduced with permission from Geng et al. (2016). (G) Schematic of a lymphatic (LV) or a venous (VV) valve. The arrow represents the direction of fluid flow. The endothelial cells of the vessel are represented in green, the endothelial cells on the upstream and downstream sides of the valve in red and yellow, respectively. The expression profiles of upstream and downstream cells are presented in the red and light green boxes, respectively. (H) SEM of a mature VV located at the opening of the external jugular vein in a newborn mouse pup. Notice the elongated valvular endothelial cells along the rim of the valve (arrows). Image reproduced with permission from Geng et al. (2016). (I) SEM of valvular endothelial cells (magenta) from an E14.5 mouse embryo, which migrate in a ‘knitting-like’ manner during VV morphogenesis in the jugular vein. Image reproduced with permission from Geng et al. (2016).

The development of the atrioventricular valves and semilunar valves starts at approximately embryonic day (E)9.5 in mouse embryos (Fig. 1A). At this stage, the heart is a simple tube consisting of the inner endocardial layer, composed of endothelial cells, and an outer myocardial layer. Signals from the myocardial cells trigger the endothelial cells to differentiate into mesenchymal (migratory) cells that delaminate into the space between the endocardial and myocardial layers and proliferate (Figs 1A,B and 2C). This process is known as the endothelial-to-mesenchymal transition (EndMT). The mesenchymal cells also secrete ECM components and the resulting structure is known as the endocardial cushion (also known as the cardiac cushion, CC; Fig. 1, Box 1) (Markwald et al., 1975). Notch, Wnt/β-catenin and TGFβ signaling pathways promote EndMT during CC formation (Armstrong and Bischoff, 2004). In contrast, VEGF inhibits EndMT in the ‘non-valve’ regions of the endocardium. In the prospective valve region, the transcription factor nuclear factor of activated T cells 1 (NFATC1) inhibits the expression of vascular endothelial growth factor (VEGF), thereby promoting EndMT (Chang et al., 2004). NFATC1 also terminates EndMT by inhibiting the expression of genes such as *Snail1* and *Snail2* (Wu et al., 2013).

Once CC formation is complete, at E12.5, the mesenchymal cells and the endocardial cells undergo coordinated morphogenesis to form the valve leaflets (Fig. 1C) (Wirrig and Yutzey, 2014). NFATC1 regulates the recruitment of neural crest cells for the elongation of cardiac valve leaflets (Wu et al., 2013). The semilunar and the tricuspid valves form three leaflets each, whereas the mitral valve forms two. Once the valve leaflets are formed, the mesenchymal cells differentiate into valvular interstitial cells in three layers (fibrosa, spongiosa and ventricularis) with distinct tensile properties (Fig. 2A) (Hinton and Yutzey, 2011; Markwald et al., 1975). These cell layers cause the cardiac valves to bend and become streamlined with respect to blood flow (Figs 1C and 2A). The presence of chordae tendineae (CT) distinguishes the atrioventricular valves from the semilunar valves (Fig. 1D). These fibrous cords connect the downstream side of the atrioventricular valves to the papillary muscles on the inner walls of the ventricle and are important for closing the valves during systole (Hinton and Yutzey, 2011).

At the end of morphogenesis, each leaflet of every cardiac valve has an upstream side that directly faces the incoming blood and a downstream side that is exposed to the outflowing blood (Figs 1D and 2A). A single layer of endothelial cells surrounds the CC. Owing to its clinical significance, most of the molecular details known about cardiac valve endothelial cells come from the aortic valve (Mongkoldhumrongkul et al., 2016), and these details are pertinent to the other valves. Evidence from dogs indicates that the upstream and downstream endothelial cells of the semilunar valves align circumferentially along the rim of the valve (Deck, 1986) (Fig. 2B). The author hypothesizes that the perpendicular alignment of valvular endothelial cells might optimize the endothelial cell response to the backflow pressure that forms during diastole. Despite their structural similarity, the endothelial cells on the upstream and downstream sides of the aortic valve have distinct molecular characteristics (Fig. 2A). For example, endothelial cells on the upstream side of the aortic valves express CX43 and KLF2 (Inai et al., 2004; Lee et al., 2006; Simmons et al., 2005), whereas endothelial cells on the downstream side of the aortic valves express TIE1, FGFR2, BMP4, PECAM1, VCAM1 and P-selectin (Porat et al., 2004; Simmons et al., 2005). Ephrin-B2, CX37, β-catenin, PROX1, FOXC2 and GATA2 are also expressed in endothelial cells on the downstream side of the aortic valves (Cha et al., 2016; Cowan et al., 2004; Inai et al., 2004; Kazenwadel et al., 2015; Simmons et al., 2005; Srinivasan and Oliver, 2011). Owing to the expression of pro-inflammatory molecules such as VCAM1 and P-selectin, the endothelial cells on the downstream side could be permissive to valvular calcification (Simmons et al., 2005). However, they also express genes such as endothelial nitric oxide synthase (*eNOS*) that could protect valves against this pathology (Simmons et al., 2005).

### Lymphovenous valves

**Fig. 3.**
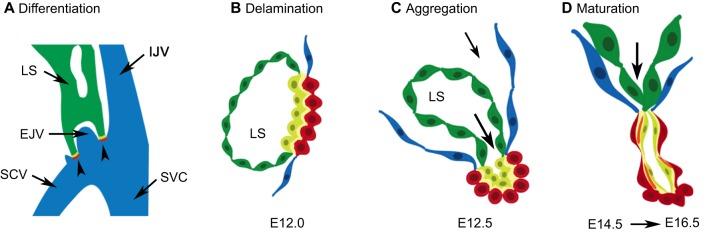
**Development of mouse lymphovenous valves (LVVs).** (A) Schematic of the junction that forms between the lymph sacs (LS; green), the internal jugular vein (IJV), external jugular vein (EJV), subclavian vein (SCV) and superior vena cava (SVC) in an E12.0 mouse embryos. The head and the heart are respectively located anterior and posterior to this location. LVVs (arrowheads) develop at the two sites of contact between the LS and the veins. (B-D) Cross-section of the junction between the LS and veins in the developing mouse embryos. (B) LVV-forming endothelial cells (LVV-ECs; red) differentiate at E12.0. Immediately after differentiation, LVV-ECs delaminate into the lumen of the vein (towards the right of this picture). Lymphatic endothelial cells (LECs) in close proximity to LVV-ECs have a distinct molecular profile relative to LECs in the LS (green), and are depicted in yellow. (C) LVV-ECs quickly reaggregate and the entire valve complex invaginates into the vein. (D) LVVs undergo further maturation by recruiting mural cells (orange) to the space between LVV-ECs and the specialized LECs. Pictures were adapted with permission from Geng et al. (2016).

LVVs are the first valves to form outside of the heart. In mice, they start forming at E12 (Geng et al., 2016; Srinivasan and Oliver, 2011) at the junction of the jugular and subclavian veins (Fig. 3A,B). LVV development begins with the differentiation of two cell types – the lymphatic endothelial cells (LECs) and LVV-forming endothelial cells (LVV-ECs) – that interact to form the LVVs. These cells upregulate the expression of the homeobox transcription factor PROX1 where the lymph sac (primitive lymphatic vessel) touches the jugulo-subclavian vein junction. Nevertheless, differences exist between these two cell types. Expression of the receptor tyrosine kinase vascular endothelial growth factor receptor 3 (VEGFR3) and CX43 is upregulated by LECs (Geng et al., 2016; Munger et al., 2016). In contrast, LVV-ECs upregulate the expression of FOXC2, GATA2 and CX37 (Geng et al., 2016; Munger et al., 2016). Additionally, integrins (α9 and α5) mediate LVV-EC–ECM and LEC-ECM interaction in the LVVs (Geng et al., 2016; Turner et al., 2014).

Immediately after differentiation, the LVV-ECs delaminate from the vein walls in the luminal orientation (Fig. 2F) (Geng et al., 2016). This process is reminiscent of the EndMT that occurs during CC formation. However, in contrast to LVV-EC delamination, endocardial cells delaminate in the abluminal direction, away from the blood flow during CC formation. LVV-ECs appear to protrude out of the venous endothelial cell layer during their delamination (Geng et al., 2016) and can withstand the force of blood flow, likely due to their association with the ECM.

The delaminated LVV-ECs dramatically elongate, reaggregate and align perpendicular to the direction of blood flow (Geng et al., 2016) (Fig. 3C), and concurrently pile up on top of each other and invaginate into the veins. An opening is created in the middle of this pile, establishing a connection between the venous and lymphatic vasculatures (Fig. 2E, asterisk) (Geng et al., 2016).

Finally, the valves recruit a few mural cells into the space between the valvular endothelial cells and the LECs (Fig. 3D) (Geng et al., 2016). At the end of the morphogenetic process, endothelial cells on both the upstream and downstream sides of LVVs are elongated and aligned perpendicular to the direction of both the venous blood flow and the lymph flow (Fig. 2E) (Geng et al., 2016).

### Venous and lymphatic valves

**Fig. 4.**
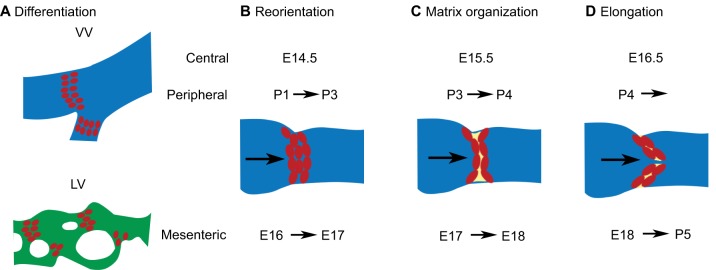
**Development of mouse lymphatic valves (LVs) and venous valves (VVs).** (A) Top: schematic sagittal section of a VV (red cells) located within the venous lumen (depicted in blue). Bottom: an LV (red cells) located within a mesenteric lymphatic vessel (depicted in green). VVs of central veins start developing at E14.5 and VVs of peripheral veins start developing at postnatal day (P)1. LVs of the mesentery start developing at E16. Images modified with permission from Bazigou and Makinen (2013). (B-D) Despite differences in their developmental time points, the morphogenesis of VVs and LVs are similar. For simplicity, a schematic of developing VVs is presented. The developmental time points corresponding to the appropriate valves are presented at the top of the pictures. The arrow within the lumen of the vessel represents the direction of fluid flow. (B) The valvular endothelial cells undergo circumferential reorientation along the rim of the vessels. (C) ECM (yellow) is organized in between the valvular endothelial cell layers. (D) The valve leaflets elongate along the direction of fluid flow to form mature valves. Images modified with permission from Bazigou et al. (2014).

VVs and LVs develop in an identical manner, sharing many similarities with LVVs (Fig. 4). In mice, VVs in the central veins (jugular and subclavian veins) start developing at ∼E14.5 and are fully formed at E16.5 (Geng et al., 2016; Munger et al., 2016). VVs in peripheral veins (such as in the saphenous veins) develop postnatally (Bazigou et al., 2011; Munger et al., 2016). LVs of the skin and mesentery start developing at around E15.5 and E16.5, respectively (Bazigou et al., 2009; Cha et al., 2016; Norrmén et al., 2009).

The differentiation of PROX1^high^; FOXC2^high^ cells is the first step of VV and LV development (Fig. 4A). Upregulated PROX1 expression seems to precede that of FOXC2, at least in the LVs (Sabine et al., 2012). These valvular endothelial cells delaminate and migrate circumferentially along the rim of the vessels. In the VVs of central veins, the VV-forming endothelial cells crisscross each other in a ‘knitting’-like process (Fig. 2I) (Geng et al., 2016). This process, called the ‘reorientation’ step because cells undergo a 90^o^ change in orientation, forms a narrow layer of valvular endothelial cells that encircles the entire circumference of the vessels (Fig. 4B) (Tatin et al., 2013). Next, the collective migration of cells constricts the lumen of the vessel while leaving a narrow aperture in the middle (Bazigou et al., 2011; Geng et al., 2016). Subsequently, integrins mediate the organization of the ECM in the valve region (Fig. 4C) (Bazigou et al., 2009), and cells along the inner edge of the circular shelf elongate in both directions and touch the vessel wall. This completes the formation of dome-shaped bicuspid valves with two commissures (Figs 2H and 4D). Finally, VVs and LVs recruit a few mural cells between the upstream and downstream endothelial cell layers (Geng et al., 2016; Kanady et al., 2011).

To summarize, valves develop in a stepwise manner. Defects in any one of these steps could result in defective valves or in valves that are susceptible to degeneration. In the following section, we discuss disease-associated genes and their mechanisms of action during valve development. We also describe the signaling pathways that regulate the expression of these genes. Readers might have already noted that most of the genes that are necessary for VV development are also required for LV or LVV development and *vice versa*. We hope to demonstrate that the role of these genes extends also to the aortic valve.

## Molecular underpinnings of valve development and disease

### Shear stress as a developmental signal

In blood vessels, shear stress is the frictional force experienced by the endothelial cells due to fluid flow. Depending on the nature of fluid flow, the cells could experience either laminar or oscillatory shear stress (Box 1). In this section we will discuss the data regarding the relationship between the nature of shear stress, valve development and valve disease.

#### Specification of valvular endothelial cells

A high-speed imaging analysis in zebrafish embryos has revealed that significant backflow occurs at the atrioventricular junction prior to valve development (Vermot et al., 2009). The expression of genes such as *klf2a*, *bmp4* and *notch1b*, which are necessary for valve formation, is enriched in this area. Importantly, reducing the reversing flow or shear stress can reduce the expression of *klf2a*, *bmp4* and *notch1b*, and abolish valve formation. Consistent with this report, KLF2 has been shown to be necessary for CC formation and for the expression of genes such as *Gata4*, *Tbx5* and *Ugdh* in mouse embryos (Chiplunkar et al., 2013).

Based on these findings, a study by Sabine et al. proposed oscillatory shear stress as being the signal that specifies LV-ECs (Sabine et al., 2012). Consistent with this model, oscillatory shear stress can enhance the expression of FOXC2, GATA2, CX37, ephrin-B2 and ITGA9 in LECs (Cha et al., 2016; Kazenwadel et al., 2015; Liu et al., 2014; Sabine et al., 2012; Sweet et al., 2015). Together, these data indicate that oscillatory shear stress is the most upstream regulator of valve specification.

#### Differentiation of aortic valve endothelial cells

As mentioned previously, the endothelial cells on the upstream and downstream sides of aortic valves have distinct molecular profiles. The upstream side of the valve faces the incoming blood; endothelial cells on this surface are exposed to high laminar shear stress. In contrast, the downstream side of the valve is exposed to turbulent and reversing flow and oscillatory shear stress (Fig. 2A). It is believed that these distinct patterns of mechanical force control the identity of the valvular endothelial cells (Chien, 2007). Results from *in vitro* flow culture models strongly support this hypothesis. Porcine aortic vascular endothelial cells were exposed to defined shear stress for 24-48 h before analysis (Dekker et al., 2002). Aortic vascular endothelial cells exposed to laminar shear stress upregulated the expression of markers such as *KLF2* and *CX43* that are enriched on the upstream side of the cardiac valves. In contrast, aortic vascular endothelial cells exposed to oscillatory shear stress expressed genes such as *SELP* that are normally expressed on the downstream side of the aortic valves.

Therefore, high laminar shear stress and oscillatory shear stress regulate the identities of endothelial cells on the upstream and downstream sides of aortic valves, respectively. In turn, endothelial cell identity is essential for inhibiting the progression of CAVD.

### Conserved polarized expression of shear-stress-responsive genes

The expression of PROX1, FOXC2, GATA2, connexins and ephrin-B2 is conserved between the aortic and vascular valves (Fig. 2A,D,G). Intriguingly, some of these molecules show side-specific expression patterns. For example, PROX1 expression is enriched on both the upstream and downstream sides of vascular valves and it is specifically expressed on the downstream side of the aortic valve (Bazigou et al., 2011; Geng et al., 2016; Sabine et al., 2012; Srinivasan and Oliver, 2011). CX43 is specifically localized to the upstream side of the aortic valves, and of VVs, LVs and LVVs (Inai et al., 2004; Kanady et al., 2011; Munger et al., 2017, 2016, 2013; Simmons et al., 2005). In contrast, CX37 expression is enriched on the downstream side of valves (Geng et al., 2016; Inai et al., 2004; Kanady et al., 2011; Munger et al., 2017, 2016, 2013; Sabine et al., 2012). Although FOXC2 is expressed in all endothelial cells, it is enriched on the downstream side of the aortic valve, VVs, LVVs and LVs (De Val et al., 2008; Geng et al., 2016; Kazenwadel et al., 2015; Munger et al., 2016; Sabine et al., 2012; Simmons et al., 2005). Likewise, although GATA2 is expressed in all endothelial cells, its expression is enriched on the downstream side of aortic valves and LVVs (Geng et al., 2016; Kazenwadel et al., 2015; Khandekar et al., 2007). In cardiac valves, the GATA2 expression domain is broader than that of PROX1 and FOXC2 (Kazenwadel et al., 2015). Whether there is any polarized expression of GATA2 in LVs and VVs is currently unknown. Finally, ephrin-B2 is expressed on the downstream side of aortic valves and VVs (Bazigou et al., 2011; Cowan et al., 2004). Owing to their important roles in vascular valve development, we speculate that PROX1, FOXC2, GATA2, CX37 and ephrin-B2 might protect the calcification-prone downstream side of aortic valves. As mentioned previously, the expression of most of these molecules is enhanced by oscillatory shear stress in LECs. In the following paragraphs we discuss these mechanisms further.

#### FOXC2

FOXC2 was the first molecule shown to be regulated by oscillatory shear stress in primary human LECs (Sabine et al., 2012), and is necessary for the expression of genes such as *CX37* (Kanady et al., 2011; Sabine et al., 2012). Oscillatory shear stress promotes an increase in cytoplasmic calcium levels in a CX37-dependent manner (Sabine et al., 2012). The mechanisms of CX37 activity during this process are not fully understood. NFATC1, a FOXC2 cofactor, is dephosphorylated by the calcium-dependent serine/threonine protein phosphatase calcineurin (Norrmén et al., 2009). Dephosphorylated NFATC1 translocates into the nucleus of oscillatory-shear-stress-exposed LECs and associates with FOXC2 (Sabine et al., 2012).

NFATC1 and FOXC2 are expressed on the opposite sides of venous valves (Munger et al., 2016). Furthermore, NFATC1 is expressed on both the upstream and downstream sides of cardiac valves (Wu et al., 2011). Therefore, the relationship between FOXC2 and NFATC1 remains to be better elucidated. It is possible that these two transcription factors interact during the early stages of valve development, as recently reported (Lyons et al., 2017).

FOXC2 and its paralog FOXC1 are both strongly expressed on the downstream side of aortic valves (Simmons et al., 2005). Deletion of both *Foxc1* and *Foxc2* in mice results in the dysplasia of CC with increased cell death in neural crest cells (Seo and Kume, 2006). However, the role of FOXC2 in CAVD is currently unknown.

#### GATA2

Flow chamber experiments have revealed that oscillatory shear stress activates GATA2 expression in primary human LECs (Kazenwadel et al., 2015; Sweet et al., 2015). In turn, GATA2 upregulates the expression of numerous molecules, including FOXC2 and ITGA9, in oscillatory-shear-stress-exposed LECs. GATA2 also directly activates PROX1 expression, presumably in an oscillatory-shear-stress-independent manner (Kazenwadel et al., 2015). Consistent with these findings, the expression of FOXC2 and PROX1 is downregulated in the valvular endothelial cells of mice lacking *Gata2* (Geng et al., 2016; Kazenwadel et al., 2015).

Whether GATA2 is involved in aortic valve development or disease is currently unknown. Two Emberger syndrome patients with mitral valve vegetations (Box 1) and embolic strokes had their symptoms attributed to defective endocardial function of GATA2 (Spinner et al., 2014). Moreover, single-nucleotide polymorphisms (SNPs) in *GATA2* are associated with coronary artery disease and atherosclerosis, diseases that frequently occur together with CAVD (Connelly et al., 2006; Muiya et al., 2014).

#### PROX1

PROX1 is the master regulator of lymphatic vascular development and a pioneer regulator of vascular valve development. Analysis of mouse embryos has revealed that PROX1 upregulation is a definitive sign that vascular valve development has begun (Bazigou et al., 2009; Norrmén et al., 2009; Srinivasan and Oliver, 2011). GATA2 is necessary for PROX1 upregulation in LVV-ECs and LV-ECs (Kazenwadel et al., 2015). Whether GATA2 regulates PROX1 expression in VVs remain unknown.

In mice, deleting a single *Prox1* allele results in severe embryonic edema (Wigle and Oliver, 1999). An analysis of *Prox1^+/−^* mouse embryos just before birth revealed that they lack LVVs, LVs and VVs (Geng et al., 2016; Srinivasan and Oliver, 2011). Oscillatory shear stress does not regulate PROX1 expression in primary human LECs (Sabine et al., 2012; Sweet et al., 2015). However, PROX1 is essential for the proper response of LECs to oscillatory shear stress (Sabine et al., 2012). LECs that lack PROX1 dramatically downregulate the expression of CX37 and VE-cadherin, and they align abnormally with respect to the direction of shear stress. Thus, PROX1 plays an important role in the shear stress response of LECs.

The mechanisms that regulate PROX1 expression in the aortic valves and whether PROX1 plays any role in CAVD are currently not known.

#### Connexins

Connexins are gap-junction molecules that regulate the transport of small molecules between adjacent cells (Kanady and Simon, 2011). Six connexin molecules oligomerize to form a hemichannel in one cell. The interaction between two hemichannels from adjacent cells forms a gap-junction channel. Gap-junction channels cluster together to form gap junctions. Three separate connexins are known to play important roles in valve development, two of which are regulated by shear stress.

##### Connexin 37

*Cx37^−/−^* mice lack VVs and have reduced numbers of LVs (Kanady et al., 2011; Munger et al., 2013). Although *Cx37^−/−^* embryos have LVV-ECs, their morphogenesis is defective and they do not invaginate into the vein (Geng et al., 2016).

CX37 expression is activated by oscillatory shear stress and by FOXC2 in LECs (Kanady et al., 2011; Sabine et al., 2012). CX37 promotes the nuclear translocation of the FOXC2 cofactor NFATC1 in LECs exposed to oscillatory shear stress (Sabine et al., 2012). This likely establishes a positive feedback loop between CX37 and FOXC2 activity. In support of this possibility, a genetic interaction has been shown to occur between mouse CX37 and FOXC2 (Kanady et al., 2015; Sabine et al., 2012). *Foxc2^+/−^* and *Cx37^+/−^* mice have normal numbers of LVs in the mesentery. However, *Foxc2^+/−^;Cx37^+/−^* mice have significantly fewer mature LVs (Sabine et al., 2012). Additionally, whereas *Cx37^−/−^* embryos have a reduced number of LVs compared to wild-type littermates, *Foxc2^+/−^;Cx37^−/−^* embryos have no LVs (Kanady et al., 2011, 2015).

CX37 is strongly expressed on the downstream side of the aortic valve (Inai et al., 2004). Whether the progression of CAVD is influenced by the loss of CX37 is currently unknown. However, *Cx37^−/−^; ApoE^−/−^* mice are more prone to high-fat-diet-induced inflammation of aortic endothelial cells and to atherosclerosis (Wong et al., 2006). CAVD and atherosclerosis are often comorbidities in human patients. Therefore, it is possible that CX37 plays a protective role in CAVD.

##### Connexin 43

CX43 is expressed on the upstream side of all the valves (Fig. 2A,D,G), and laminar shear stress activates its expression in blood endothelial cells (Butcher and Nerem, 2007; Inai et al., 2004). In contrast, CX43 expression is downregulated by oscillatory shear stress in both blood endothelial cells and LECs (Butcher and Nerem, 2007; Sabine et al., 2012).

When fed a Western diet, *Ldlr^−/−^* mice, which lack the low-density-lipoprotein receptor (LDLR) that is necessary for cholesterol endocytosis, develop atherosclerosis (Wong et al., 2003). In contrast, *Cx43^+/−^; Ldlr^−/−^* mice fed a Western diet are resistant to atherosclerosis, indicating that CX43 plays a pro-atherogenic role in mouse blood vessels (Wong et al., 2003). Its downregulation on the downstream side of the aortic valve could therefore fulfill an atheroprotective role in the vasculature (Mongkoldhumrongkul et al., 2016). As mentioned previously, *Tie2-Cre;* *Cx43^f/f^* mice are viable (Liao et al., 2001; Theis et al., 2001). Whether these mice are better protected from CAVD needs to be explored.

##### Connexin 47

Expression analysis has revealed that CX47 widely colocalizes with CX43 on the upstream side of LVs and VVs during their early development (Kanady et al., 2011; Munger et al., 2016). At later stages, CX47 expression continues to colocalize with CX43, but it is restricted to fewer cells. CX47 is not expressed in LVVs (Munger et al., 2016). Whether CX47 is regulated by shear stress and whether it plays any role in aortic valve development or disease is unknown.

In summary, connexins are important for valve development. However, their mechanisms of action in valve formation and function are not fully understood. As mentioned previously, CX37 is necessary for oscillatory-shear-stress-induced NFATC1 activation. Whether any other signals are regulated by connexins remains to be investigated. Connexins could also play non-channel roles, such as in regulating cell-cell interaction and the production of the vasodilator nitric oxide (Kanady and Simon, 2011; Meens et al., 2015; Pfenniger et al., 2010).

### EPHB4 and ephrin-B2

The receptor tyrosine kinase EPHB4 was originally identified as a vein-specific marker and its membrane-bound ligand ephrin-B2 was identified as an artery-specific marker (Gerety et al., 1999). These molecules have mutually exclusive expression patterns and produce repellent signals that promote the segregation of arteries and veins (Gerety et al., 1999). Signals downstream of EPHB4 are known as forward signaling and those downstream of ephrin-B2 as reverse signaling.

Expression of ephrin-B2 is enhanced by oscillatory shear stress in a GATA2-independent manner (Sweet et al., 2015). However, the relationship between ephrin-B2 and shear stress has not been explored further. Ephrin-B2 is required for the development of LVs and VVs (Bazigou et al., 2011; Makinen et al., 2005). Loss of the cytoplasmic domain of ephrin-B2 results in thickened cardiac valves (Cowan et al., 2004). Therefore, ephrin-B2 reverse signaling might be important to inhibit the progression of CAVD.

There is an interesting controversy regarding the role of ephrin-B2 reverse signaling during LV development. Mutating the phosphorylated tyrosine residues to phenylalanine in the cytoplasmic tail of ephrin-B2 results in the arrest of LV morphogenesis (Makinen et al., 2005). However, deletion of the entire cytoplasmic tail seemingly does not affect LV morphogenesis (Zhang et al., 2015). It is proposed that the tyrosine-to-phenylalanine mutations inhibit the cytoplasmic-to-cell-surface translocation of the ligand and precludes EPHB4 forward signaling (Cowan et al., 2004).

Antibodies that specifically promote EPHB4 signaling were recently shown to rescue the LV defects in mice lacking ephrin-B2 (Zhang et al., 2015), highlighting this as a potential therapeutic approach to treating the LVV and LV defects of patients with EPHB4 mutations. Furthermore, if EPHB4 or ephrin-B2 function in CAVD, signal-modulating antibodies could play a role in the fight against this devastating disease.

### ITGA9

ITGA9 expression is enhanced by oscillatory shear stress in a GATA2-dependent manner (Sweet et al., 2015). NOTCH1 is also necessary for the expression of ITGA9 in LVs (Murtomaki et al., 2014). ITGA9 can heterodimerize with integrin-β1 (α5β1) and associate with ECM proteins, such as with the EIIIA-domain-containing fibronectin (Fn-EIIA), SVEP-1 and EMILIN1 (Bazigou et al., 2009; Danussi et al., 2013; Karpanen et al., 2017; Morooka et al., 2017). Indeed, *Fn-EIIA^−/−^* mice recapitulate the phenotype of *Itga9^−/−^* animals (Bazigou et al., 2009). *Svep-1*- and *Emilin1*-null mice also have valve defects (Danussi et al., 2013; Karpanen et al., 2017; Morooka et al., 2017).

In summary, mechanosensitive molecules that are important for valve development and morphogenesis are beginning to be identified. In the following section we will discuss the mechanotransduction mechanisms that activate the expression of these molecules.

### Mechanotransduction mechanisms during valve development

We are beginning to understand the mechanisms that endothelial cells use to sense the various patterns of fluid flow and translate them into chemical signals. Here, we discuss the mechanosensory molecules that function in mechanotransduction and in lymphatic vascular maturation or LV development.

#### Wnt/β-catenin signaling

We recently showed that oscillatory shear stress promotes the stabilization and nuclear translocation of β-catenin in primary human LECs (Cha et al., 2016), and that oscillatory-shear-stress-enhanced FOXC2 expression depends on β-catenin activity. Wnt/β-catenin signaling also enhances PROX1 expression in LECs in an oscillatory-shear-stress-independent manner. β-catenin associates with the regulatory elements of *PROX1* and *FOXC2* in primary human LECs (Cha et al., 2016). Consistent with these findings, the conditional deletion of β-catenin from the LECs of mice results in the loss of LVVs, VVs and LVs (Cha et al., 2016).

Expression of β-catenin is enriched on the downstream side endothelial cells of aortic valves (Simmons et al., 2005). Consistent with this report, the deletion of β-catenin in these cells abolished FOXC2 expression (Cha et al., 2016). These results suggest that Wnt/β-catenin signaling transduces oscillatory shear stress to activate FOXC2 expression in valvular endothelial cells. As mentioned previously, GATA2 enhances the expression of PROX1 and FOXC2 in valvular endothelial cells (Kazenwadel et al., 2015). As such, it will be important to test whether GATA2 synergizes with β-catenin to enhance PROX1 and FOXC2 expression.

#### Syndecan-4

In blood endothelial cells, platelet and endothelial cell adhesion molecule 1 (PECAM-1), VEGFR2 and VE-cadherin form a mechanosensory complex that mediates flow response (Tzima et al., 2005). VEGFR3 is also a component of this mechanosensory complex in blood endothelial cells (Coon et al., 2015). Whether VEGFR2, VEGFR3 and VE-cadherin are necessary for flow response in LECs remains unknown. However, PECAM-1 was recently shown to act in parallel with syndecan-4 during mouse lymphatic vessel and LV morphogenesis (Wang et al., 2016). The lymphatic vessels of *Sdc4^−/−^* mice are hyperproliferative and hyperbranched, and the LV-forming endothelial cells do not reorient properly during the circumferential elongation process. These defects are more severe in *Sdc4^−/−^;Pecam-1^−/−^* embryos. Mechanistically, syndecan-4 knockdown in LECs affects their ability to align correctly with respect to the direction of laminar shear stress. This defect is due to the overexpression of planar cell polarity (PCP) protein VANGL2 in the cells with reduced syndecan-4. Knockdown of VANGL2 rescues the flow-induced alignment of LECs with reduced syndecan-4 (Wang et al., 2016).

VANGL2 and CELSR1 are transmembrane proteins that function in the Wnt/PCP pathway, which coordinates cell polarity across the tissue plane (Devenport, 2014). During LV morphogenesis, VANGL2 and CELSR1 localize to cell junctions, where they inhibit the accumulation of VE-cadherin to prevent the stabilization of adherens junctions (Tatin et al., 2013). Thus, syndecan-4 acts as a vital link between the laminar shear stress and PCP pathways. Presumably, VANGL2 and CELSR1 destabilize cell junctions during LV morphogenesis, whereas syndecan-4 stabilizes them once the morphogenetic process is complete. How or whether syndecan-4 mechanistically interacts with PECAM-1 to regulate VANGL2 expression is currently unknown, as is whether syndecan-4 is important for the oscillatory shear stress response.

In summary, the Wnt/β-catenin and Wnt/PCP pathways play important roles in endothelial cell shear response during valve formation. Recently, the calcium channel ORAI1 was reported to be necessary for laminar-shear-stress-induced inhibition of Notch signaling and lymphatic vascular growth (Choi et al., 2017). Whether this pathway operates during valve development also remains to be investigated.

### Limitations of the shear stress model

Endothelial cells from opposite sides of the aortic valve maintain their identities in culture even when cultured under identical conditions for prolonged periods (Simmons et al., 2005). This suggests that the side-dependent phenotypes of these cells are likely to be dictated by developmental and local environmental factors, as well as by shear stress and hemodynamics.

Endothelial cells derived from the aorta (the major artery originating from the left ventricle) are commonly used to gain insights about the shear response of aortic valve endothelial cells. One study evaluated the different responses of aortic vascular endothelial cells and aortic valve endothelial cells to flow (Butcher et al., 2004, 2006). The authors determined that cardiac valve endothelial cells align perpendicular to laminar shear stress in the flow chamber, thus recapitulating the *in vivo* phenotype of those cells. However, vascular endothelial cells align parallel to laminar shear stress. In addition, at least 10% of genes are differentially expressed between these two cell types in response to laminar shear stress, and the signaling pathways that regulate the endothelial cell response to flow are also distinct. The authors noted that caution was required when extrapolating findings from vascular endothelial cells to valvular endothelial cells (Butcher and Nerem, 2007).

The same caution is also warranted when using LECs to describe LV development. Although LECs exposed to oscillatory shear stress assume a round morphology, LV-ECs are elongated *in vivo* and are perpendicularly organized with respect to fluid flow (Geng et al., 2016; Sabine et al., 2012). It will be important to determine the mechanisms that regulate the LV-EC identity, which in turn dictates their shear response. Additionally, how the oscillatory and laminar shear stress responses are integrated during valve development remains to be understood.

**Fig. 5.**
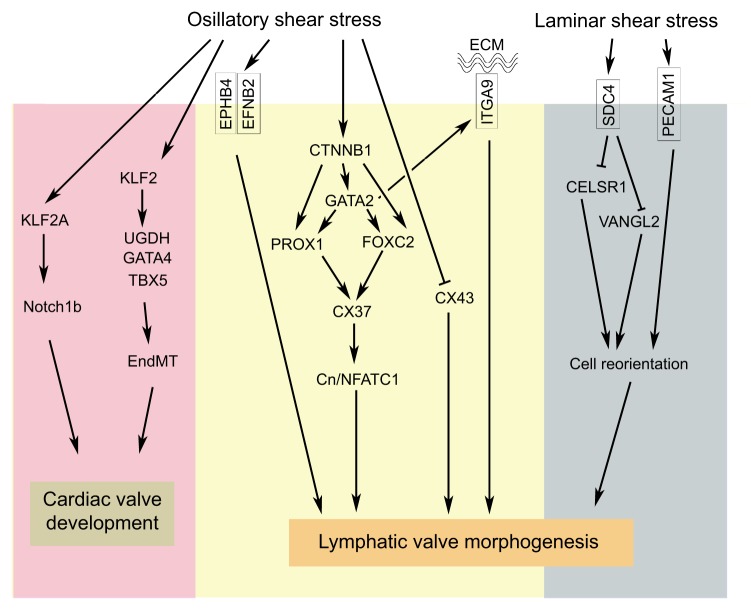
**An integrated model for valve development.** A model of the various molecules that regulate valve morphogenesis and their functional relationships. Oscillatory shear stress activates *Klf2* expression and its downstream target genes during cardiac valve development in zebrafish and mice. During lymphatic valve development, oscillatory shear stress enhances the expression of multiple molecules, such as ITGA9, ephrin-B2 (EFNB2), GATA2, FOXC2, CX37 and β-catenin (CTNNB1). Oscillatory shear stress also antagonizes CX43 expression. β-catenin is upstream of GATA2 and FOXC2. Shear-stress-activated GATA2 is upstream of FOXC2 and ITGA9. PROX1 expression is not regulated by oscillatory shear stress. However, both GATA2 and β-catenin could enhance PROX1 expression in an oscillatory-shear-stress-independent manner. CX37 regulates NFATC1 activity through the calcineurin (Cn) signaling pathway. Laminar shear stress inhibits the planar cell polarity molecules VANGL2 and CELSR1 through syndecan-4 (SDC4). PECAM1 acts in parallel with SDC4 to regulate the reorientation of valve-forming endothelial cells.

In summary, shear stress plays an important role in valve development. However, approaches to study the response of valvular endothelial cells to shear stress need to be further refined. Whenever possible, valvular endothelial cells should be used for cell culture experiments. However, because these cells are difficult to obtain and maintain, novel approaches, such as transdifferentiation of vascular endothelial cells to valvular endothelial cells, need to be developed. Importantly, the molecular and cellular mechanisms that are currently unrelated to the shear-stress response should not be ignored (see Box 2). Our current understanding of the mechanisms of valve development is presented in Fig. 5.

**Box 2. Proteins involved in shear-stress-unrelated mechanisms that regulate vascular valve development**

**Angiopoietin-2:** a ligand for the receptor tyrosine kinase TIE2; promotes the expression of FOXC2 (Dellinger et al., 2008; Morooka et al., 2017).

**BMP9:** signals through ALK1 to promote the expression of neuropilin-1, FOXC2, ephrin-B2 and CX37 (Levet et al., 2013).

**CDK5:** a cyclin-dependent kinase that phosphorylates FOXC2 and promotes its activity (Liebl et al., 2015).

**ITGA5:** mediates cell-ECM interaction during valve morphogenesis (Turner et al., 2014).

**Notch signaling:** regulates the expression of ITGA9 (Murtomaki et al., 2014).

**RASA1:** inhibits the Ras signaling pathway and prevents apoptosis (Lapinski et al., 2017).

**Semaphorin-3A:** activates the plexin-A1–neuropilin-1 complex during left-ventricle morphogenesis (Bouvree et al., 2012; Jurisic et al., 2012).

**SVEP1:** ECM protein that enhances the signals mediated by angiopoietin-2 (Karpanen et al., 2017; Morooka et al., 2017).

**TIE1:** an orphan tyrosine-kinase receptor that promotes the expression of PROX1 and FOXC2 (Qu et al., 2015).

## Conclusions and perspectives

We end this Review with a few final thoughts regarding the challenges and opportunities for valve research. Owing to large variations in the penetrance and the time of disease onset, the treatment strategies may have to be custom designed for the patients. To achieve this goal, we need to understand the reasons for the variable penetrance and etiology of the disease. We speculate that some of the shear-responsive genes could be used as diagnostic markers. Next, once the target patients are chosen, the treatment strategies may have to be tailored to their specific needs. Target-specific drugs could be the best option for treating the disease that starts *in utero*. Once again, the molecules that we discussed in this article could be important targets for drug development. Surgical repair/replacement of valves could be a feasible option for patients whose disease starts postnatally. Because there are hundreds of LVs and VVs in the human body, identifying the most critical valves could help in efficiently targeting this treatment. Furthermore, there are only four LVVs in mammals and several studies (mouse and human) have indicated that these valves could be defective in lymphedema. Therefore, we need to explore whether repairing these valves could cure or ameliorate lymphedema.

Valve replacement surgery is already available for aortic valves. As discussed above, this approach has its limitations and a better understanding of the biology of aortic valve endothelial cells is needed for advancing the treatments. Simmons et al. used human gene expression microarrays to identify genes that are differentially expressed between the upstream and downstream sides of porcine heart (Simmons et al., 2005). Since then, both microarray and RNA-seq technologies have improved tremendously. Thus, repeating this experiment using advanced tools might reveal additional side-specific genes. Mouse models could demonstrate the significance of these genes during aortic valve development and disease. It will be important to develop conditional mouse models to specifically delete genes on the upstream or downstream sides of aortic valves without affecting the vascular valves. This will also require the creation of new Cre lines.

In conclusion, in the past 10 years, creative experiments that are built on models put forward by aortic valve biologists have revealed that shear forces regulate the expression of genes that are necessary for vascular valve development. Intriguingly, these genes are also expressed in the aortic valves, thus revealing a previously unanticipated commonality between the aortic valves and vascular valves. We hope that this proposal will stimulate discussion between cardiac valve and vascular valve researchers, and accelerate the search for cures for valve diseases.
